# Epstein-Barr Virus Genome Deletions in Epstein-Barr Virus-Positive T/NK Cell Lymphoproliferative Diseases

**DOI:** 10.1128/jvi.00394-22

**Published:** 2022-05-25

**Authors:** Wiyada Wongwiwat, Benjamin Fournier, Irene Bassano, Amr Bayoumy, Claudio Elgueta Karstegl, Christine Styles, Ray Bridges, Christelle Lenoir, David BoutBoul, Despina Moshous, Bénédicte Neven, Teru Kanda, Rhys G. Morgan, Robert E. White, Sylvain Latour, Paul J. Farrell

**Affiliations:** a Section of Virology, Department of Infectious Disease, Imperial College London, London, United Kingdom; b Laboratory of Lymphocyte Activation and Susceptibility to EBV Infection, INSERM UMR1163, Institut Imagine, Paris, France; c Department of Pediatric Immunology, Hematology, and Rheumatology, Necker-Enfants-Malades Hospital, APHP, Paris, France; d Université de Paris, Paris, France; e Department of Clinical Immunology, Saint-Louis Hospital, APHP, Paris, France; f Division of Microbiology, Tohoku Medical and Pharmaceutical University, Sendai, Japan; g School of Life Sciences, University of Sussex, Brighton, United Kingdom; University of Arizona

**Keywords:** Epstein-Barr virus, T lymphocyte, NK cell, chronic active EBV, hemophagocytic lymphohistiocytosis

## Abstract

The main target cells for Epstein-Barr virus (EBV) infection and persistence are B lymphocytes, although T and NK cells can also become infected. In this paper, we characterize the EBV present in 21 pediatric and adult patients who were treated in France for a range of diseases that involve infection of T or NK cells. Of these 21 cases, 5 pediatric patients (21%) and 11 adult patients (52%) were of Caucasian origin. In about 30% of the cases, some of the EBV genomes contain a large deletion. The deletions are different in every patient but tend to cluster near the BART region of the viral genome. Detailed investigation of a family in which several members have persistent T or NK cell infection by EBV indicates that the virus genome deletions arise or are selected independently in each individual patient. Genome sequence polymorphisms in the EBV in these T or NK cell diseases reflect the geographic origin of the patient and not a distinct type of EBV (the 21 cases studied included examples of both type 1 and type 2 EBV infection). Using virus produced from type 1 or type 2 EBV genomes cloned in bacterial artificial chromosome (BAC) vectors, we demonstrate infection of T cells in cord blood from healthy donors. Our results are consistent with transient infection of some T cells being part of normal asymptomatic infection by EBV in young children.

**IMPORTANCE** EBV contributes to several types of human cancer. Some cancers and nonmalignant lymphoproliferative diseases involving T or NK cells contain EBV. These diseases are relatively frequent in Japan and China and have been shown sometimes to have deletions in the EBV genome in the disease cells. We identify further examples of deletions within the EBV genome associated with T or NK cell diseases, and we provide evidence that the virus genomes with these deletions are most likely selected in the individual cases, rather than being transmitted between people during infection. We demonstrate EBV infection of cord blood T cells by highly characterized, cloned EBV genomes and suggest that transient infection of T cells may be part of normal asymptomatic infection by EBV in young children.

## INTRODUCTION

In addition to being the main cause of infectious mononucleosis, Epstein-Barr virus (EBV) infection contributes to a range of human cancers (1); these are mainly lymphomas and carcinomas derived from B lymphocytes or epithelial cells, the two main target cell types for normal adult EBV infection *in vivo* (2, 3). In these cancers, the viral genome is usually intact (4). EBV infection of T or NK cells is now also recognized to be involved in various additional EBV-positive T/NK lymphoproliferative diseases (5). These diseases include extranodal NK/T cell lymphoma (ENKTL), aggressive NK cell leukemia/lymphoma (ANKL), systemic EBV-positive T cell lymphoma of childhood (STCL), EBV-positive T/NK hemophagocytic lymphohistiocytosis (HLH), and chronic active EBV infection (CAEBV), which is subdivided into a systemic form and two cutaneous forms, including hydroa vacciniforme (HV). These diseases often arise in children but can also develop in adults. ANKL and ENKTL are overt malignant diseases. Some cases of leiomyosarcoma also carry EBV in the tumor cells.

CAEBV and NK/T lymphomas associated with EBV are more frequent in Asian populations, particularly in Japan and China (6–8), than in western countries. Detailed analysis of the virus DNA has shown that deletions in the EBV genome are present in a significant proportion of the cases of these T cell and NK cell infection diseases. The deletions most often occur in or near the BART microRNA (miRNA) region of the EBV genome, potentially giving some insight into the mechanism by which EBV may contribute to the diseases. Some similar cases of CAEBV with deletions in the EBV genome have recently been investigated in the United Kingdom (9), and relapse after treatment has been studied in CAEBV patients from the United States, Canada, Israel, and New Zealand (10).

Although EBV-infected T cells are not found in normal adult carriers of the virus, children in Africa have been shown frequently to have some EBV-infected T cells (11), and certain strains of EBV (particularly type 2 EBV) have been found to infect T cells in culture (12). The main EBV receptor for B cell infection is CD21, also known as complement receptor 2 (CR2). CD21 was shown to be involved in EBV infection of a T cell line (13). HLA class II molecules can also play an important role in EBV infection of B and T cells (14–16), and HLA class II molecules can be sufficient for EBV infection of B cells without CD21 (17). In contrast to the preference for type 2 EBV infection in cell culture, most of the T and NK cell disease cases that have been studied have type 1 EBV, reflecting the prevalent EBV type in those populations. The reasons why certain type 2 strains of EBV were found to be more effective at T cell infection in cell culture are unclear at present; it might be due to the specific virus genomes, but infectivity can also be influenced by membrane proteins from the different cell hosts used to produce the virus stock. To study T cell infection, it would thus be valuable to be able to produce EBV from bacterial artificial chromosome (BAC)-cloned virus genomes, with the same cell line used as host for production of all virus stocks.

In this paper, we analyze the EBV DNA present in a set of patients treated in France who all have EBV in their T or NK cells, associated with their disease. Many of these cases have been reported previously (18), and some have been investigated for inherited immune deficiencies by whole-exome sequencing. One patient was found to be a carrier of biallelic loss-of-function mutations in the *TNFSFR9* and *PIK3CD* genes (5, 19). We show that the EBV in these patients with infected T or NK cells is classified normally according to its geographic origin and is not a distinct type of EBV. Our detailed analysis confirms the presence of deletions in the EBV BART genome region in some cases. Results from a family with several patients exhibiting EBV-infected T or NK cells in their blood (one patient with CAEBV and two asymptomatic cases) support the conclusion that the EBV genome deletions in T and NK cell diseases arise independently in the patients. Although EBV normally infects B lymphocytes in the blood, we show that a small fraction of CD3^+^ T cells from cord blood (but not adult peripheral blood) can be infected in cell culture with BAC-cloned type 1 or type 2 EBV. This will allow future analysis of viral genome sequences that may influence T cell infection and persistence by EBV.

## RESULTS

### EBV in T or NK cell infections is not a distinct type of EBV.

Blood samples were obtained from 21 patients treated in France (samples were collected at Institut Imagine, Paris, France) who had various diseases in which EBV was present in T or NK cells, as described previously (18) (Table 1). The patients’ diseases included ANKL, HV, systemic CAEBV with inflammatory bowel disease (IBD), STCL, and disseminated smooth muscle tumors. In each case, there was EBV in the T or NK cells. Five patients were Caucasian, but the majority (*n* = 15) were from Africa, including 11 from North Africa. One patient and his family were from Pakistan (Table 1).

**TABLE 1:** Summary of patient characteristics^*a*^

Patient	Sex	Age at onset (yr)	Nationality	Case in reference 18	Disease	Cell type with EBV
1	F	13	Caucasian (French)		Systemic T/NK cell CAEBV (myositis, nephrotic syndrome)	CD3^+^
2	M	43	North African	3.8	Nasal NK cell disseminated ENTKL/T cell lymphoma, circulating EBV-infected NK cells	CD3^−^ CD16^+^
3	M	5	Caucasian (French)	5.2	HV	TCRγδ^+^
4	M	4	Caucasian (French)		STCL	CD8^+^
5	M	40	North African or Turkish	5.3	HV	TCRγδ^+^
6	M	14	Sub-Saharan African	6.3	Systemic T/NK cell CAEBV, IBD	NK (circulating), CD3^+^ (gut)
7	M	11	Sub-Saharan African	6.6	Systemic T/NK cell CAEBV, large-vessel vasculitis	CD3^low^ CD4^−^ CD8^−^ TCRγδ-
8	M	7	North African	6.4	Systemic T/NK cell CAEBV, mucocutaneous lymphoproliferation	CD4^+^ TCRγδ^+^
9	F	4	North African		STCL	CD8^+^
10	F	35	Caucasian (French)	6.9	Systemic T/NK cell CAEBV, indolent tumor on the floor of the mouth	CD4^+^
11	F	3	North African	6.1	Systemic T/NK cell CAEBV, mucocutaneous lymphoproliferation	CD8^+^, NK
12	M	14	Pakistani		Systemic T/NK cell CAEBV (recurrent fever and hepatomegaly)	CD8^+^
13	M	24	Caucasian (French)	3.3	Liver transplantation, persistent peripheral EBV load after rituximab, IBD	CD8^+^
14	M	35	Sub-Saharan African		Systemic T/NK cell CAEBV, nodular cutaneous vasculitis with EBV-T cell infiltration	CD8^+^
15	F	12	Caucasian (French)	5.1	HV	TCRγδ^+^
16	M	22	North African	6.5	Systemic T/NK cell CAEBV, large-vessel vasculitis	TCRγδ^+^
17	M	52	North African	S5	Systemic T/NK cell CAEBV, chronic liver disease, splenomegaly	CD4^+^ TCRγδ^+^
18	F	25	Nigeria		*GATA2* deficiency, condylomatosis, medullar and blood NK cell lymphoproliferation	NK, CD4^+^
19	M	11	French Guiana		Systemic T/NK cell CAEBV, IBD, fulminant HLH acutization	NK
20	M	31	North African		ANKL	NK
21	F	9	North African		Disseminated EBV-positive smooth muscle tumors, chronic elevated blood EBV PCR	CD19^+^ CD8^+^
12S	F		Sister of patient 12		No apparent disease	T/NK
12F	M		Father of patient 12		No apparent disease	T/NK

a EBV associated smooth muscle tumors have recently been associated with TNFSF9/CD137 ligand deficiency (42).

**FIG 1:**
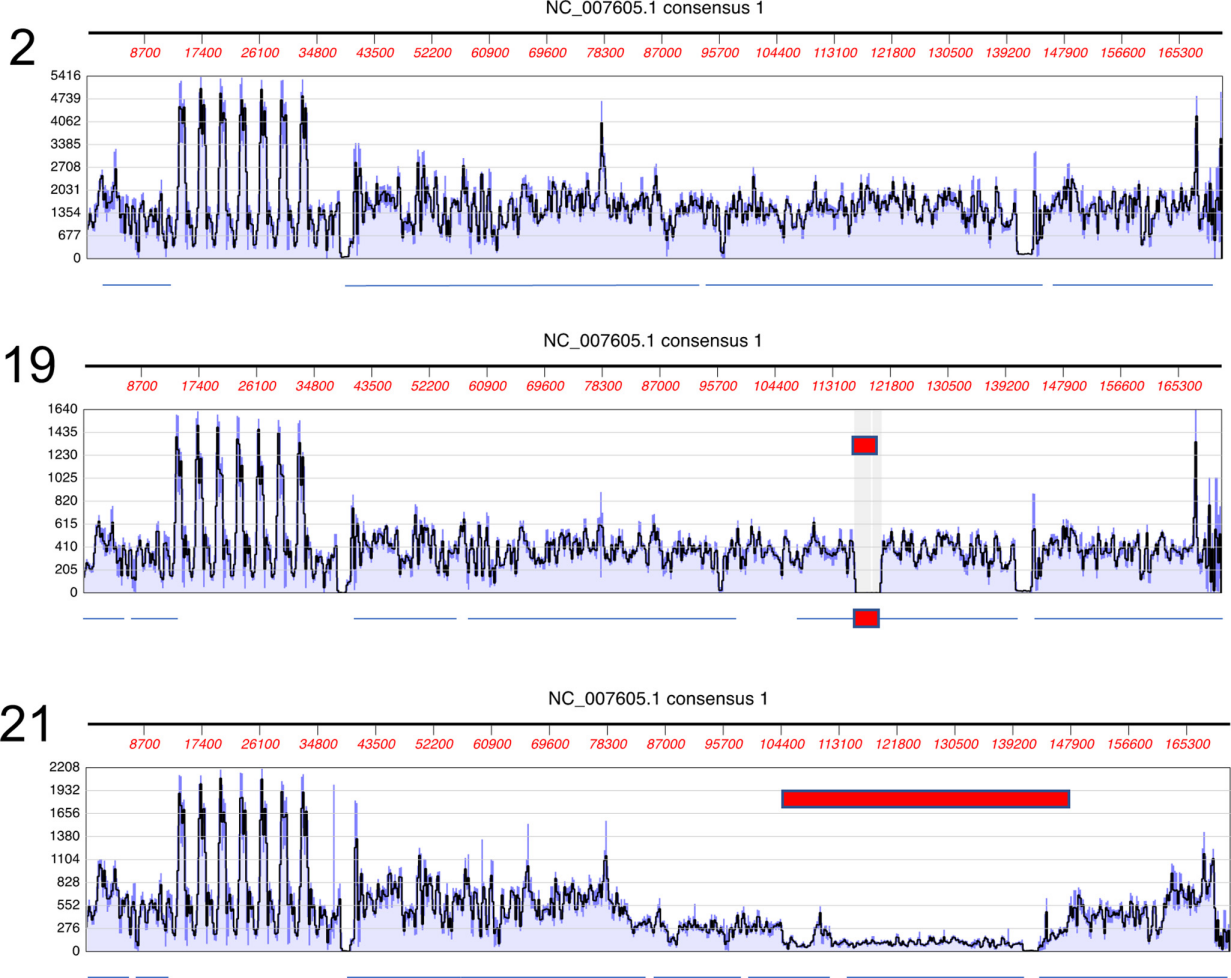
Examples of EBV sequence read depth and contig maps. EBV sequence reads were aligned to the reference type 1 (GenBank accession number NC007605) or type 2 (GenBank accession number NC009334) genome, and read depths are displayed in MacVector. The contigs obtained from the SPAdes assembly are represented by the lines beneath each panel. The deletions in the EBV genome are shown by red blocks. Systematic overrepresentations or underrepresentations of reads in some parts of the genome in all samples are due to variations in enrichment probe efficiency and sequence repeat arrays in the virus genome. Data for all samples analyzed are presented in Fig. S1 in the supplemental material.

The EBV DNA in the patient blood samples was sequenced using the target enrichment and Illumina sequencing methods described previously (20). EBV genome sequence reads were mapped with the Burrows-Wheeler aligner (BWA) reference-based assembly method to type 1 or type 2 EBV genomes (GenBank accession numbers NC007605 or DQ279927 [strain AG876]), allowing the resulting EBV genome sequences to be classified as type 1 or type 2 EBV based on the similarity of their Epstein-Barr nuclear antigen 2 (EBNA2) and EBNA3 genes to the reference sequences (Table 2). As in previous studies (20, 21), SPAdes *de novo* assemblies of the sequence reads were also made, and the major EBV contigs were then substituted into the relevant BWA sequence to produce consensus EBV genome sequences (GenBank accession numbers MT648642 to MT648662). The regions of the genomes derived from the *de novo* assembly contigs are underlined in Fig. 1 and in Fig. S1 in the supplemental material.

**TABLE 2:** Summary of EBV DNA sequence results^*a*^

Patient	EBV type	Deletion positions (numbering as in GenBank accession no. NC007605)	Notes	GenBank accession no.
1	T2			MT648642
2	T1			MT648643
3	T1	139808–147575	No nucleotides in gap	MT648644
4	T1			MT648645
5	T1			MT648646
6	T2	138664–147924	No nucleotides in gap; deletion is positions 139492–149408 in AG876 (GenBank accession no. DQ279927) numbering	MT648647
7	T1			MT648648
8	T1			MT648649
9	T1			MT648650
10	T1			MT648651
11	T1			MT648652
12	T1	123556–126276	No nucleotides in gap	MT648653
12		127936–151063	1 nt in gap (A)	
13	T1	99367–147053	28 nt in gap (ACGTCTTGGTCGCGACCAAGACAGTCAT)	MT648654
14	T1			MT648655
15	T1			MT648656
16	T2			MT648657
17	T1			MT648658
18	T1			MT648659
19	T1	116576–120526	No nucleotides in gap	MT648660
20	T1			MT648661
21	T1	104712–147410	Also position 163099–110807 junction, inverted	MT648662
12S (sister of patient 12)		135908-139664 del	European EBV	
12F (father of patient 12)			Pakistan EBV	

a Deletion position numbers shown are the nucleotides that are deleted. T1 is type 1 EBV, T2 is type 2 EBV.

Some of the samples contained mixtures of both full-length viral genome and a viral contig with a unique deletion. Selected examples of this can be seen in Fig. 1, in which sample 2 has the standard read depth pattern with no deletion. In sample 19, the virus genome deletion is almost complete (marked in red), and sample 21 has a partial deletion with some full-length genome also present. The complete set of read maps is shown in Fig. S1, which shows samples 3, 6, 12, 13, and 21 to have localized partial reductions in sequence read depth. Because the deletion in sample 19 was almost complete, its GenBank sequence (GenBank accession number MT648660) lacks the deleted nucleotides, but the other samples had sufficient wild-type EBV reads present to give a complete genome in the sequence deposited in GenBank (accession numbers are listed in Table 2).

**FIG 2:**
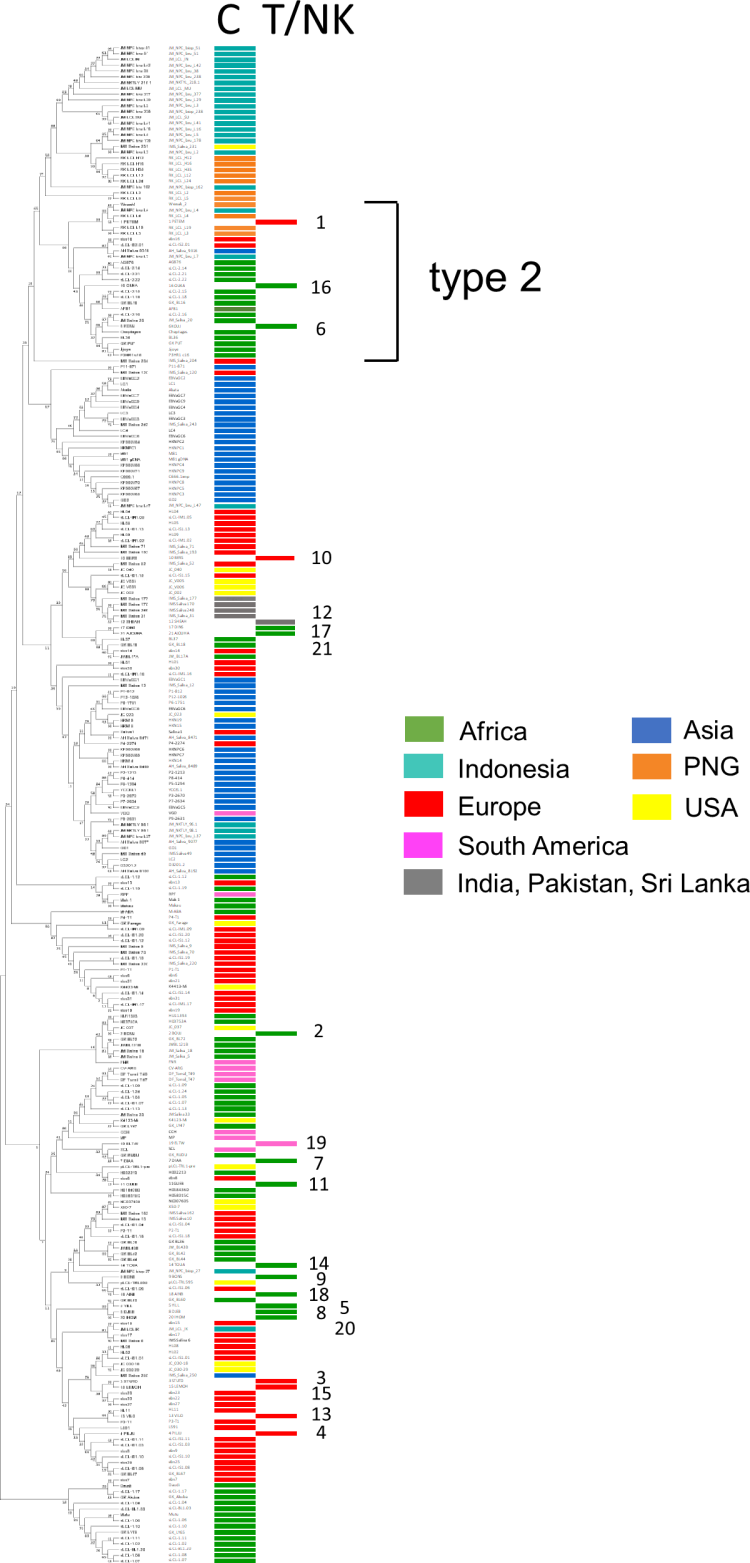
Combined phylogenetic tree of 241 (41) current (C) and new (T/NK) EBV genome sequences from patients 1 to 21 reported in this study (Table 1), showing that the new sequences are distributed according to their geographic origins. Current sequences were analyzed previously (21). The cluster of type 2 EBV genomes is indicated. An enlarged version of the same data (in which the strain names can be read) is shown in Fig. S2 in the supplemental material.

To test whether the genome sequence of EBV found in T or NK cell infections differs systematically from the standard virus found in B cells, the EBV genomes from the 21 new samples were compared to 241 EBV genome sequences we analyzed previously (21) that did not have T or NK cell infection. Those samples originated mostly from infected B cells or epithelial cells, but some had been sequenced directly from saliva. In a whole-EBV-genome phylogenetic tree, the 21 T or NK cell infection EBV genomes segregated according to their type 1 or type 2 status and their geographic origin (Fig. 2). A high-resolution version of the same phylogenetic tree in which the names of individual strains can be read is shown in Fig. S2. The 21 T or NK cell EBV sequences did not cluster separately from the B cell and epithelial cell EBV isolates; therefore, the EBV present in these T cell or NK cell infections appears to be the standard virus, although some of the viral genomes present in these diseases have a sequence deletion.

We previously used single-nucleotide polymorphism (SNP) counting (22) in multiple-sequence alignments and principal-component analysis (23) to identify positions of SNPs that are diagnostic of the EBV from specific geographic regions. Such analysis separates African, European, Asian, and Indonesian groups, but we have not previously been able to identify SNPs characteristic of EBV from the Indian subcontinent region. The phylogenetic tree shown in Fig. S2 has a cluster of 5 adjacent strains that were obtained in Europe or Asia but are known to be from people from India, Pakistan, or Sri Lanka (or with that immediate ancestry). A set of SNPs unique to this India/Pakistan/Sri Lanka group of 5 sequences was identified (see Fig. S3) using the procedures described previously (23).

In the phylogenetic tree (see Fig. S2), 2 of the 3 HV cases (samples 3 and 15) clustered adjacent to each other. Both were from Caucasian French subjects, but the third HV case (patient 5, described as North African or Turkish) clustered separately with the African EBV strains; therefore, this pattern is most likely to be due to geographic similarity rather than disease association.

### Deletions in EBV genomes in diseases with T or NK cell infections frequently affect the BART transcript region and can be markers for disease.

**FIG 3:**
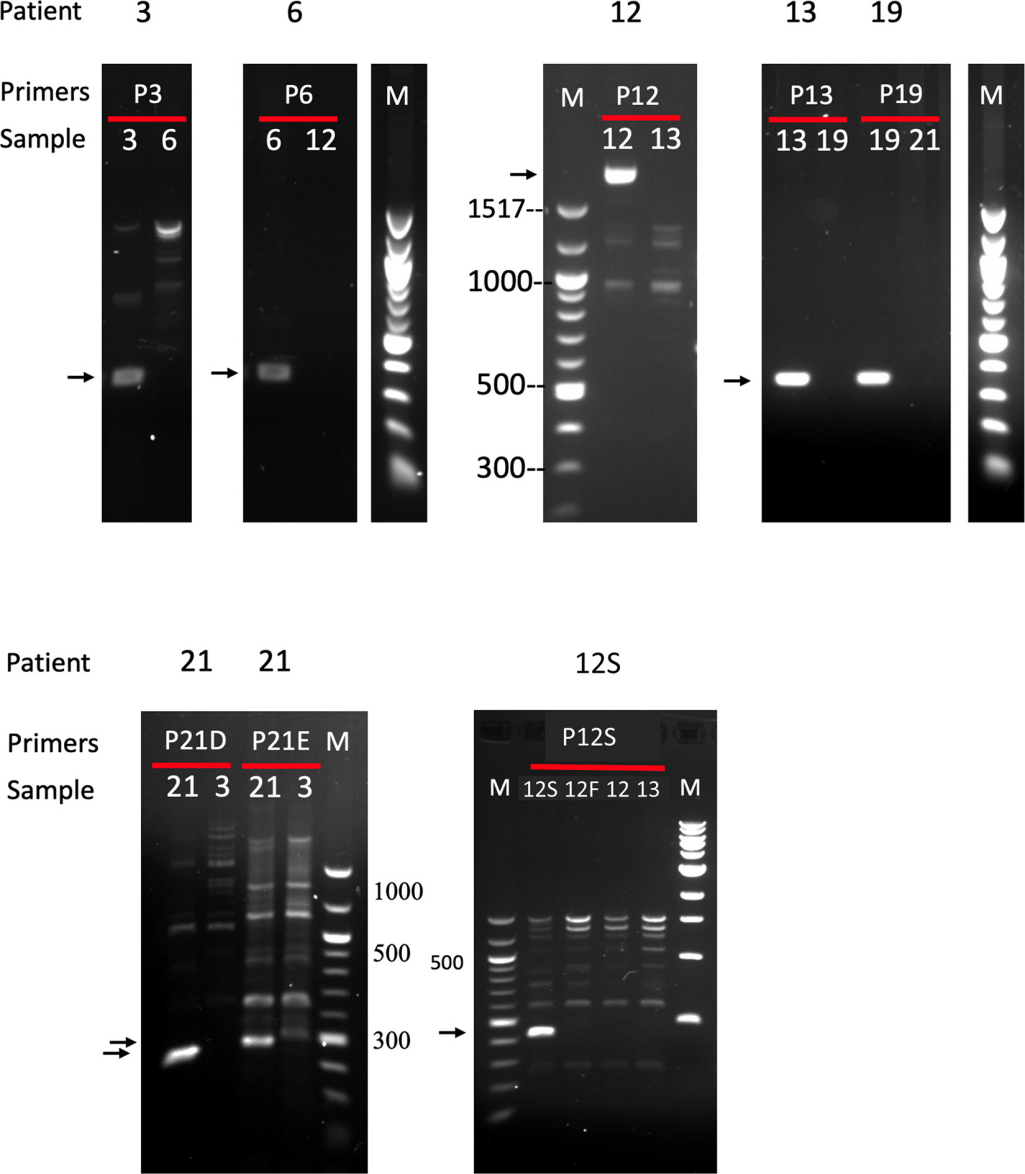
PCR amplification products across EBV genome deletions. Primer pairs (P3, P6, etc. [listed in Fig. S4 in the supplemental material]) specific for each patient’s EBV deletion were used for PCR amplification of patient sample DNA across the deletion points deduced from DNA sequencing results. In each panel, the specific primers are used with the relevant patient DNA sample and another patient DNA sample (as a negative control). Specific products marked with an arrow were subsequently eluted and sequenced to confirm the deletion points. Coelectrophoresed DNA size markers (M) are indicated.

**FIG 4:**
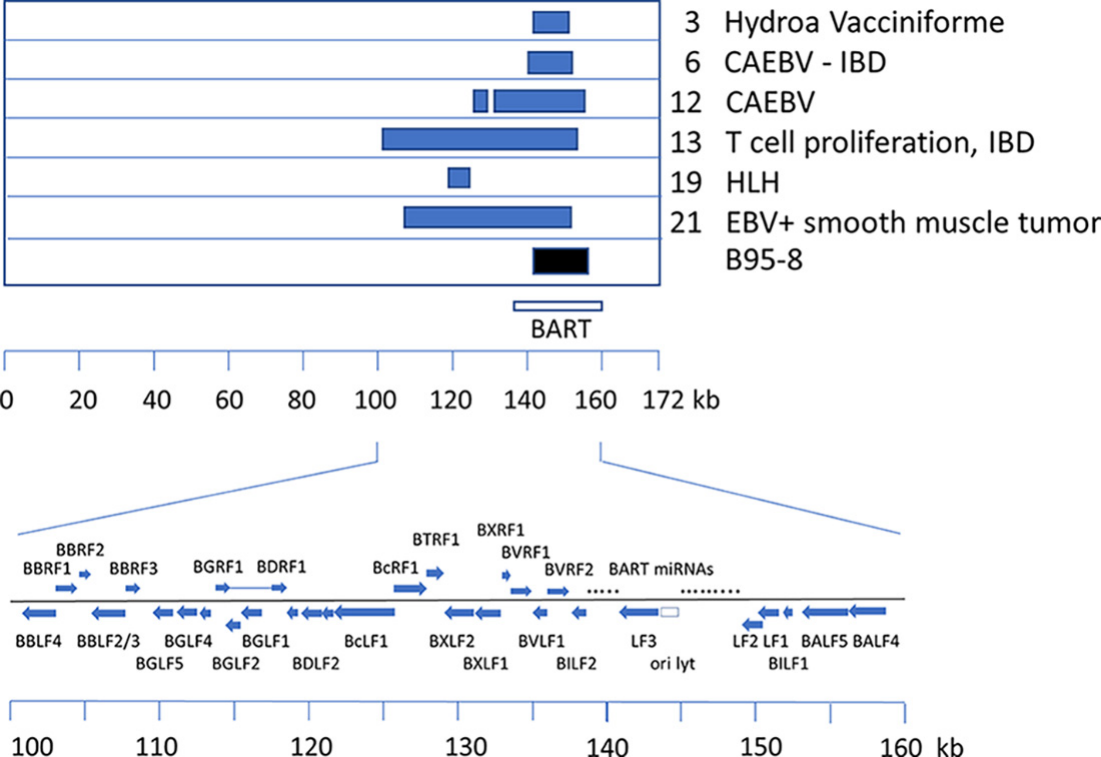
Summary of locations of EBV genome deletions. Positions of deletions are shown above a scale corresponding to the map of the reference EBV genome (GenBank accession number NC007605). The location of the BART RNA transcript region is marked, and the layout of viral genes in the part of the EBV genome affected by the deletions is illustrated.

Previous studies of CAEBV and NK/T cell lymphoma cases in Japan and China identified deletions in the viral genome in about 30 to 40% of cases analyzed (6–8). The read depth maps of the reference-based assemblies of EBV sequences from our 21 patients in France indicated that several of these samples might also have deletions in the EBV genomes (Fig. 1; also see Fig. S1). Examination of the sequence reads in the BWA assemblies suggested boundaries of the deletions, and PCR primers that would amplify across each deletion point were designed (primer sequences are listed in Fig. S4). Specific PCR products unique to each deletion that spanned the deletion points in each virus tested were obtained (Fig. 3). These were sequenced and confirmed the precise deletion points, also identifying any novel sequence that might be inserted at the breakpoint. This also confirmed that the discontinuities in the EBV sequence were internal deletions in the viral genome and did not represent points of integration into the cell chromosome. Cases 3, 6, 13, and 19 had single unique deletions (coordinates are listed in Table 2). Case 12 had two adjacent deletions, about 1.6 kb apart (Table 2). Sample 21 had a large deletion but also had a separate inversion junction, joining two parts of the EBV genome. The deletions are summarized in Fig. 4, relative to the reference EBV genome (GenBank accession number NC007605) scale. As reported previously for the Asian CAEBV and NK/T cell lymphomas (6–8), the BART locus was frequently affected by the deletions. Our data support and extend those observations with examples of T cell infections in HV, IBD, and HLH. A similar region of the genome is deleted in B95-8, the widely studied prototypical laboratory strain of EBV (Fig. 4). Although a small fraction of the EBV in peripheral blood of these patients would likely be in B lymphocytes (as expected for normal persistence of the virus), most of the EBV was in the T or NK cells listed in Table 1, as determined by the EBV-encoded small RNA (EBER) flow cytometry method (18). As indicated in Table 1, patient 21 had disseminated EBV-positive smooth muscle tumors but also had EBV-infected cells in her peripheral blood. EBER flow cytometry showed that 2.2% of CD19^+^ B cells and 3.3% of CD8^+^ T cells were EBV positive in this patient. At this stage, we do not know whether the EBV genome deletion found in patient 21 was present in one or both of these infected cell types.

For samples that did not have deletions identified in Table 2, the sequences of the BART miRNA region were examined for SNPs in the miRNA sequences that might represent mutations, as an alternative way of losing BART miRNA function. There were occasional SNPs but very few, and there was no consistent pattern. Also, no SNPs were detected in the BART miRNAs in patient 19, whose EBV deletion was distant from the BART region of the genome. Therefore, there was no evidence of loss of miRNA function in these samples from the actual miRNA sequences, although the possibility of changes in the BART RNA primary transcript affecting miRNA expression could not be excluded by this analysis. Most likely, the EBV genome variations are probably not the only factor driving the EBV-positive T/NK cell diseases.

Once a deletion has been identified in the EBV of a T/NK cell disease, PCR across the deletion point could potentially be used as a case-specific marker of disease, as discussed recently (9), although the level of deleted variant might vary with time. Quantitative PCR analysis of a repeat blood sample from patient 13 (obtained 27 months after the first sample) showed a similar level of EBV deletion PCR product, relative to the EBNA2 gene (used as a marker of all EBV genomes present in the sample), but a 5-fold lower level of EBV overall, perhaps reflecting fewer disease cells in the blood at the second sampling.

### Different EBV genome deletions in one family suggest that the deletions arise within the individual.

**FIG 5:**
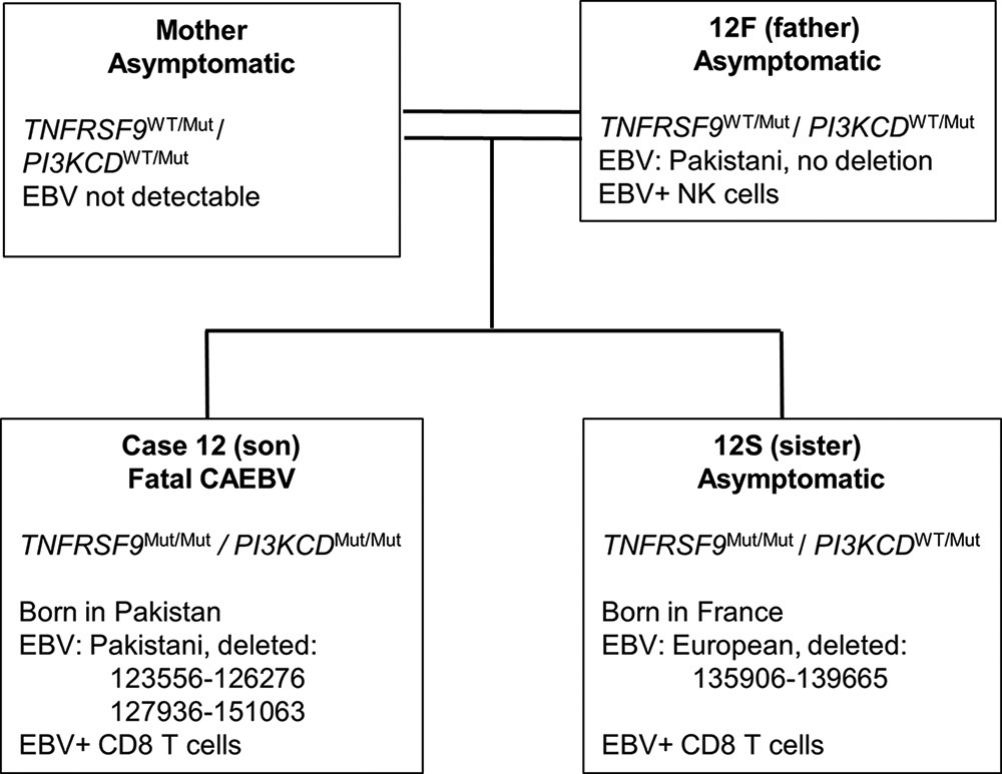
Summary of case 12 family genetics and disease information (19) with EBV genome results. CD137 is also known as TNFRSF9 or 4-1BB.

Although the EBV genome deletions tend to cluster around the BART miRNA region, the deletion boundaries observed so far are different in every case, suggesting that they arise independently. They might arise in the individual through low-level replication errors being selected in the T or NK cells, or they might perhaps be transmitted when a person is infected by EBV. Three of the samples whose EBV was analyzed came from the same family (Table 1), i.e., patient 12 (EBV-positive CD8^+^ T cells), the father of patient 12 (EBV-positive NK cells), and the sister of patient 12 (EBV-positive CD8^+^ T cells). Both patient 12 and his sister (patient 12S) exhibited high blood loads of EBV (5 × 10^6^ to 6 × 10^6^ copies/mL) persistent over years. The father (patient 12F) was recently found to have some circulating EBV-positive NK cells, but at various samplings he had either no or low blood loads of EBV (3 × 10^6^ to 4 × 10^6^ copies/mL). Data from this family (summarized in Fig. 5) suggest that the deletions arise in the individual. Both parents in this consanguineous marriage had heterozygous mutations in the *TNFRFS9* (encoding the CD137/4-1BB protein) and *PIK3CD* genes (19). These two siblings of their four children were homozygous null for *TNFRFS9* (Fig. 5). The boy (patient 12) also had a homozygous loss-of-function *PIK3CD* mutation and suffered fatal symptomatic CAEBV, while his sister (patient 12S) is still asymptomatic (and is heterozygous for the *PI3KCD* mutation). Both parents were from Pakistan and their son was born in Pakistan, but the family subsequently moved to France and the daughter was born in France. Different deletions were found in the EBV genomes present in the son and daughter (Fig. 5 and Table 2), but these were not detected in the blood of the father (patient 12F) by sequencing or by PCR specific for those deletions. EBV DNA could not be detected in the mother’s blood. The EBV genome sequences of the father and son matched the India/Pakistan/Sri Lanka consensus sequence at the geographic diagnostic SNPs, but the EBV genome of the daughter matched the European EBV at those nucleotide positions. Thus, the daughter must have acquired her EBV from outside her immediate family, but she still carried EBV with a unique genome deletion and had EBV-positive T/NK cells detectable in her blood. This implies that persistence of circulating EBV-positive T cells in her blood was promoted by the inherited immunodeficiency but her EBV genome deletion arose or was selected in her, since her EBV did not appear to be derived from her Pakistani parents or brother. The results also indicate that persistent EBV-positive T or NK cells may arise only with specific combinations of several factors; the asymptomatic father did not have complete CD137 and PIK3CD deficiency or an EBV deletion, but his asymptomatic CD137-deficient daughter carries EBV with a deletion in its genome, presumably in her CD8^+^ T cells. That deletion was different from the EBV deletion in her symptomatic brother (who had both CD137 and PIK3CD deficiencies).

### Cord blood T cells can be infected *in vitro* by type 1 and type 2 EBV.

T cells from adults generally lack the CD21 receptor for EBV infection, but most T cells from cord blood and from the blood of young children are very early thymic emigrants and are now known to express CD21 (24). A preference for type 2 EBV infection of adult T cells in cell culture (12) and in Kenyan children (11) has been reported. Since most of our patients with T or NK cell infection diseases had type 1 EBV, we sought to clarify the ability of type 1 or type 2 EBV to infect cord blood T cells.

**FIG 6:**
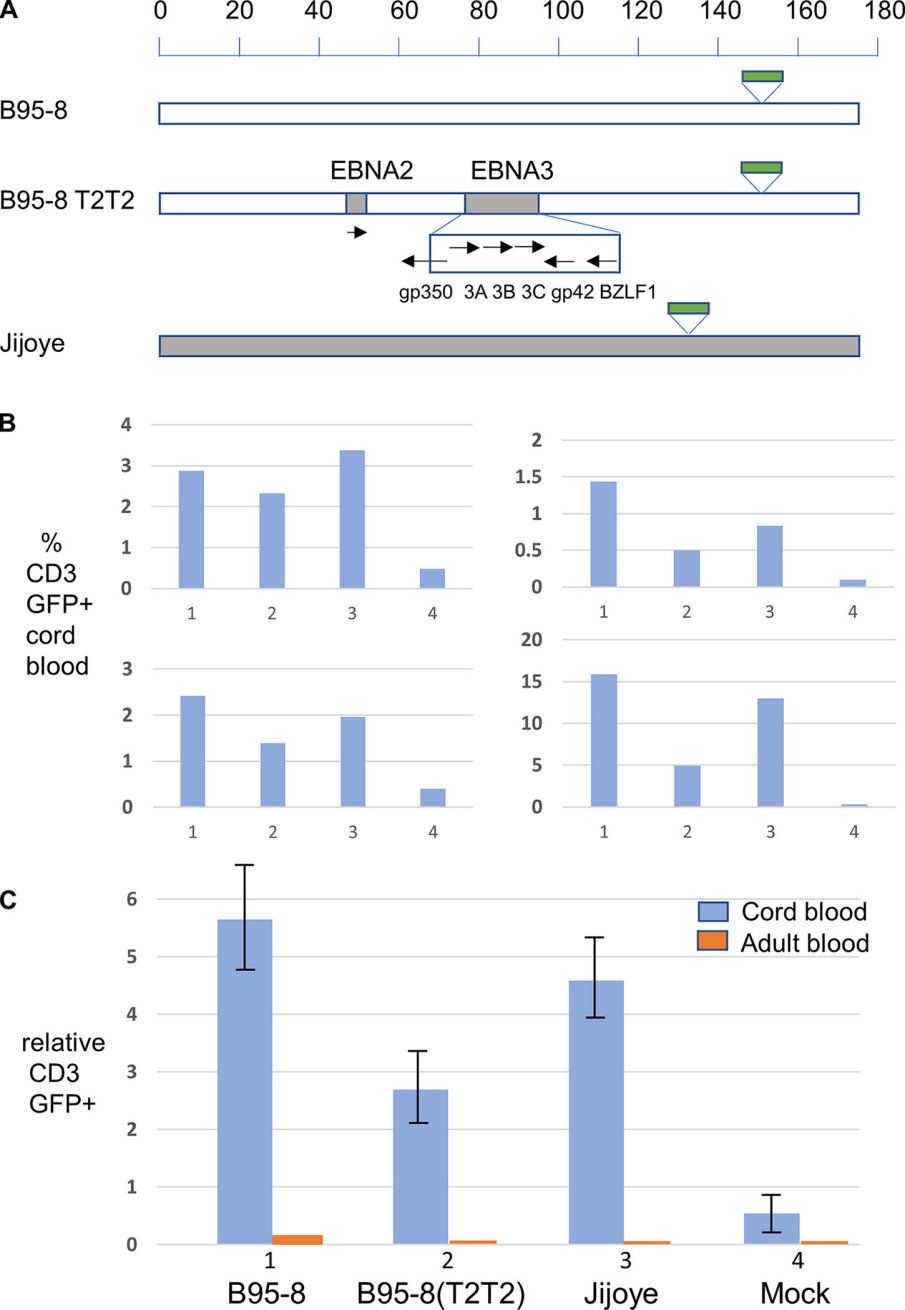
(A) Summary of BAC-cloned EBV genomes used in this study on a scale in kilobases, showing the position of the GFP gene insertion in the BAC vectors (green bar). B95-8 is EBV type 1, and Jijoye is EBV type 2. The shaded parts of the B95-8 T2T2 genome indicate the substitution of type 2 sequence into the B95-8 BAC clone. Arrows show the EBV genes in the substituted regions and their orientations (3A, 3B, and 3C indicate EBNA3A, EBNA3B, and EBNA3C, respectively). (B) T cell infection of CD3^+^ T cells in cord blood mononuclear cells by B95-8 BAC, B95-8 T2T2 BAC, or Jijoye BAC EBV strains expressing GFP. Infection levels determined by flow cytometry are shown as the percentage of CD3^+^ cells that are GFP positive in four cord blood cell infection experiments using different cord blood samples (1, B95-8; 2, B95-8T2T2; 3, Jijoye; 4, mock [no EBV added]). (C) Relative infection levels of cord blood T cells (blue bars). The average normalized numbers of GFP-positive cells in the CD3^+^ cell gate using data from the 4 biological replicates in panel B are shown with standard deviations. Equivalent values for infection of adult T cells (orange bars) are from 3 biological replicates with the same virus stocks.

In order to do this, we cloned the type 2 EBV strain Jijoye, which was previously reported to infect T cells (12), into an F factor-based BAC plasmid. Briefly, the BAC was cloned into the BamHI V region of the EBV genome, adapting a previously published EBV-cloning strategy (25) to improve the stability of the EBV BAC (see Fig. S5). EBV was produced from 293 cells stably carrying the Jijoye EBV BAC (type 2), the B95-8 EBV BAC (type 1), or the same B95-8 EBV in which both the EBNA2 locus and the EBNA3 locus (including flanking parts of gp350 and gp42) had been exchanged for type 2 EBV sequence (Fig. 6A). All of these BAC-cloned viruses contain the green fluorescent protein (GFP) gene as a reporter to titrate the virus infectivity measured in green Raji units (26). Because they are produced in the same cell line background, there should be no differences in the virus stocks due to the host cells.

Cord blood mononuclear cells were infected at a multiplicity of infection (MOI) of 0.1 green Raji units per cell and analyzed by flow cytometry 8 days after the addition of virus. Using GFP expression to indicate infection, most of the infected cells were CD19^+^ B cells (data not shown), but GFP expression from the virus was also observed in some CD3^+^ cells, consistent with infection of T cells (Fig. 6B). Although the efficiency of T cell infection varied substantially between donors (from 1% to 15% of T cells), the pattern of infection efficiency by the viruses was consistent between the donors (Fig. 6B). Averaging the normalized results of 4 separate experiments with different cord blood samples did not show a significant difference in the relative levels of T cell infection between the B95-8 (type 1) and Jijoye (type 2) EBV (Fig. 6C). The infectivity of the BAC-cloned B95-8 EBV was not improved by substituting the EBNA2 and EBNA3 loci with type 2 EBV sequence (Fig. 6C). The lower relative GFP-positive CD3^+^ cell value with this virus (Fig. 6C) was not due to a deficiency in this recombinant virus, because similar analysis of CD20^+^ B cells at the same time point in the same cord blood experiments showed that an average of 24.3% of the B cells were GFP positive with B95-8 (T2T2), compared to 19.4% with the B95-8 type 1 EBV and 15.3% with the Jijoye EBV. In contrast to the infection of cord blood cells, no significant infection of T cells from adult peripheral blood was observed with the same virus stocks (Fig. 6C).

## DISCUSSION

The diseases involving EBV infection of T or NK cells that we studied in this work develop in children and young adults (5). In most of the cases we analyzed, the EBV genome appears to be intact; however, in 6 of the 21 patients we studied and in 1 of 2 asymptomatic individuals, there was a deletion in some of the EBV genomes. At about 30%, this is a frequency similar to that observed in studies of CAEBV and lymphoma in China and Japan (6–8). As observed previously, these deletions are in the part of the EBV genome between the EBNA1 open reading frame and the BART miRNA cluster. The boundaries of the deletions appear to be different in every case, but they frequently include part of the BART miRNA region, perhaps suggesting a connection between loss of BART miRNA expression and disease. Similar EBV genome deletions also occur in certain EBV-positive B cell lymphomas (particularly diffuse large B cell lymphoma) with similar frequencies (7), suggesting that mutation in this genomic region favors EBV persistence (and consequent malignancy of EBV-positive clones), rather than being involved in a specific T/NK cell tropism.

Because the precise EBV deletion boundaries are different in every case, they seem to have developed or been selected within the individual, rather than there being transmission of mutant EBVs. Here, we report a family with inherited immunodeficiencies in CD137 and PIK3CD, three members of which have T or NK cell-associated EBV. This would be a likely circumstance in which to observe transmission of a mutant EBV strain if it contributed to EBV establishment in T and NK cells. However, we established not only that EBV mutations were unique to each child but also that the EBV in the two children came from different sources; one was a South Asian EBV strain, and the other was a European strain. The simplest explanation for these observations is that occasional errors in replication or recombination of the virus genome generate defective genomes, which are not normally observed because they are less fit to complete the normal asymptomatic virus life cycle. They might, however, offer growth and survival advantages to the infected cell and thus might be amplified or selected in disease. This seems to imply an active role for the deleted genome in the diseases, but we cannot tell from this analysis whether the cells with deleted EBV genomes also contain full-length EBV genomes.

In addition to the patients with EBV infection of T cells, several of the patients we studied had EBV abnormally present in NK cells. There has been no indication of CD21 expression on NK cells or direct infection of NK cells in cell culture. One possibility would be that a common precursor of T and NK cells in hematopoietic development might be infected, and this would then differentiate to give the EBV-infected NK cells (6, 27). Alternatively, NK cells have recently been found to be able to receive CD21 and EBV from B cells by trogocytosis (28), and this might explain how they occasionally become infected. Also, CD21 might not be a prerequisite for infection of NK cells, which also express HLA class II when activated, potentially allowing infection (17).

Previous studies focused on the ability of type 2 EBV strains to infect T cells but, using cloned viral genomes, we found that both type 1 EBV (B95-8) and type 2 EBV (Jijoye) were able to infect T cells from cord blood but neither virus infected adult T cells. This is consistent with most disease cases studied having type 1 EBV (which is the main type of EBV worldwide) but occasional cases having type 2 EBV. The main sequence differences between type 1 and type 2 EBV strains are in EBNA2 and the region including the EBNA3 genes (21). When the EBNA2 and EBNA3 region sequences from type 2 EBV were recombined into the type 1 B95-8 (giving B95-T2T2), no enhancement of infection was observed, relative to the type 1 B95-8 EBV. It should be noted that B95-8 EBV has a deletion in the BART miRNA region of the viral genome similar to that found in some of the disease cases, possibly reducing its ability to suppress interferon induction (29), but there is no indication in the data shown here of greater infection of cord blood T cells by type 2 EBV in cell culture, compared to the type 1 B95-8 EBV.

Studies of EBV infection using peripheral blood mononuclear cells (PBMCs) from adults or B cells from adenoids emphasized the important role of the EBV BART miRNAs in immune evasion, avoiding inhibitory effects of interferons and inflammatory responses induced by the infection (29). Immune evasion thus appears to be a major function of the BART miRNAs in EBV, and this was supported by studies of cancer cell lines (30). The infection of cord blood T cells we observed in cell culture was transient but the EBV infection of T cells in the patients appears to persist, perhaps because of the host genetic background, such as the CD137 and PIK3CD deficiencies in patient 12, as proposed previously for the familial cases of T or NK cell infections described in this paper (18, 19). Perhaps the impairment of BART miRNA expression caused by some of the deletions would favor persistence in T or NK cells in the disease situation. In fact, only about 30% of cases had a deletion in the EBV genome, and one of those deletions was not in the BART region; therefore, there are likely to be several different mechanisms by which the persistence of EBV could develop in T cells. Loss of BART miRNA expression could be one of those potential mechanisms, in combination with host genetic background determinants.

The widely accepted model for natural EBV infection of people has EBV transmitted in saliva, replicating briefly in oral epithelial cells, and then infecting B lymphocytes in the tonsils or other lymphoid tissue in the oropharynx. In the B lymphocytes, it traffics through B cell activation and selection in lymph nodes, eventually getting to its site of latent persistence in memory B cells (2). There is extensive evidence to support this, but the scheme was largely deduced by identifying which cells are infected in adults carrying EBV (or can be infected in cell culture using blood cells from adults). However, primary EBV infection normally occurs in young children, usually in the first few years of life. This infection in young children usually happens with no symptoms of disease, and it is the process for which EBV has evolved. In contrast, primary infection of adults often causes infectious mononucleosis, in which T and NK cells are strongly activated and express high levels of HLA class II, which could potentially explain the EBV-infected T or NK cells occasionally observed in tonsils during this disease (31–34). The hematology and immunology of young children differ from those of adults in several ways. Particularly relevant to this is the significant expression of CD21 (the main receptor for EBV infection) on naive T cells or recent thymic emigrant T cells in the blood of young children (24). CD21 is not normally expressed on the T cells in the blood of adults (24). Although B cells are the main target cells for EBV infection in young children, there is clear evidence from studying children in Africa that EBV infection of their T cells (in addition to their B cells) is frequently observed (11) and we have shown that cord blood T cells can be infected by EBV. Therefore, it may be necessary to modify the standard model for the cell biology of natural EBV infection in infants and young children to include some degree of transient T cell infection.

## MATERIALS AND METHODS

### Patient samples and DNA analysis.

DNA was extracted from patient blood samples using the QIAamp DNA blood maxikit (Qiagen). Written informed consent was obtained from all human participants in this study in accordance with the Helsinki Declaration, local legislation, and ethical guidelines from the Comité de Protection des Personnes de l’Ile de France II, Hôpital Necker-Enfants-Malades (Paris, France). PCR analysis of EBV deletions used the Q5 Hot Start High-Fidelity DNA polymerase kit (New England Biolabs).

EBV DNA target enrichment and sequencing were as described previously (20). The reference-based sequence assembly used BWA version 0.7.17-r1188, and *de novo* assembly was with SPAdes version 3.13.0-Linux.

### BAC-cloned EBV strains.

The B95-8 type 1 EBV BAC clone p2089 (35) was a kind gift from H. J. Delecluse and W. Hammerschmidt. T2T2 B95-8 EBV, in which the EBNA2 and EBNA3 loci have been replaced by corresponding sequences from AG876 type 2 EBV, was created by sequentially recombining the EBNA3 sequence and then the EBNA2 sequence into a version of p2089 lacking the EBNA3 locus called E3KO (36), using a RecA-based bacterial recombineering strategy (37). A 22.1-kb region of the AG876 type 2 EBV genome containing the EBNA3 locus was captured in a hybrid yeast artificial chromosome (YAC)/BAC vector by transformation-associated recombination (TAR) cloning in yeast spheroids, and then a SalI restriction fragment excised from this clone (positions 77328 to 93838 of the AG876 sequence [GenBank accession number DQ279927]) spanning the EBNA3 region was cloned into a recombineering vector and recombined with E3KO. The resulting EBV recombinant genome was validated by restriction digestion and sequenced after EBV hybrid enrichment (20) of the DNA from the virus producer cell line. This confirmed that the type 2 EBNA3 region DNA sequence replaced positions 78020 to 91439 of the type 1 EBV (numbering as in GenBank accession number NC007605). Then, plasmid p121 containing the type 2 EBNA2 gene was recombined as described previously (38) to give a B95-8 virus backbone with type 2 EBNA2 and EBNA3 genes. The type 2 EBNA3 region in this virus includes the sections of gp350 and gp42 that differ between type 1 EBV and type 2 EBV (21) and the Zp V3 promoter (21) found in type 2 EBVs (Fig. 6A). EBNA2 was the only gene replaced by the recombination at the EBNA2 locus.

The type 2 Jijoye EBV genome was cloned into a BAC vector that contained GFP and hygromycin expression cassettes inserted between the BVRF1 and BVLF1 open reading frames, adapting a published procedure (25, 39). A capture vector linearized between overlapping homology regions in the BVRF1/BVLF1 region of the EBV genome was transfected into EBV-positive B cells alongside a CRISPR/Cas9 construct that cuts the recombination site in the EBV genome, and then cells were selected with hygromycin for 1 month prior to recovery of the EBV-BAC genome into bacteria. The previously published capture vector (25, 39) contains overlapping regions within the upstream and downstream homology arms flanking the transgenes. Initial attempts to clone Jijoye EBV with this vector produced a clone that was unstable (via recombination between these repeated regions) during large-scale DNA preparations. Therefore, we altered the nucleotide sequences of the overlapping regions of the upstream and downstream homology arms while keeping the sequences of BVRF1 and BVLF1 intact (see Fig. S5A in the supplemental material) and produced three stable clones of the Jijoye EBV strain. Validation of these clones by restriction digestion and pulsed-field gel electrophoresis (see Fig. S5B) showed a reduced number of W repeat units in clone C16 and also a deletion in clone L3, which Nanopore sequencing identified as encompassing the promoter driving GFP but leaving an intact EBV genome in clone L3. Nanopore sequencing of clone C4 showed 99.8% identity to the published Jijoye genome sequence (GenBank accession number LN827800) in the unique regions that were sequenced by both methods; therefore, this BAC clone was used to make virus-producing 293 cell clones as described previously (40).

Virus production from the HEK293 cell lines containing BAC EBV genomes was induced by transfection of a 1:1:1 ratio of plasmids expressing gB (BALF4), BZLF1, and BRLF1 as described (26). Virus titers were determined by infection of Raji cells, to measure green Raji units (26).

### EBV infections.

Human cord blood was obtained with informed consent from healthy mothers at full term undergoing elective caesarean sections at the Royal Sussex County Hospital, with approval from the Brighton & Sussex University Hospitals NHS Trust, the East of England-Essex Research Ethics Committee, Human Research Authority, and Health and Care Research Wales (approval number 18/EE/0403). Mononuclear cells were separated using Ficoll-Hypaque (Merck Millipore, Dorset, UK) and samples with ≥80% viability (deduced from trypan blue exclusion) were included in the study.

With consent from NHS Blood and Transplant (NHSBT), leukocyte reduction system cones from healthy donors were used to isolate adult PBMCs. The cone cells were layered over Ficoll-Paque Plus (GE Healthcare), and the mononuclear cells were isolated by density gradient centrifugation.

Blood mononuclear cells were infected with recombinant EBV at a MOI of 0.1 green Raji unit per cell in the presence of 1 μg/ml cyclosporin A. On day 8 after infection, the viability of infected cells was determined using the LIVE/DEAD Fixable Aqua dead cell stain kit (number L34957; Thermo Fisher Scientific). The cells were then washed and surface stained with antibodies specific for CD3 (phycoerythrin [PE]-Cy7 conjugated, number 557851; BD Pharmingen), CD20 (R-phycoerythrin [RPE] conjugated, number R7013; Dako), and CD21 (allophycocyanin [APC] conjugated, number 561357; BD Pharmingen). The stained cells were analyzed on a Becton, Dickinson LSRFortessa cell analyzer, and data were analyzed with FlowJo software (version 10.8.1).

### Data availability.

EBV genome sequences reported in this paper (listed in Table 2) have been deposited in GenBank under accession numbers MT648642 to MT648662.
