# Supplementary material for: Epstein-Barr Virus Genome Deletions in Epstein-Barr Virus-Positive T/NK Cell Lymphoproliferative Diseases
Source: J Virol. 2022 May 25;96(12):e00394-22. doi: 10.1128/jvi.00394-22 (PMC9215254; doi:10.1128/jvi.00394-22)
Supplement: Supplemental file 1 — Fig. S1 and S3-S5 and legends. Download jvi.00394-22-s0001.pdf, PDF file, 1.5 MB [file jvi.00394-22-s0001.pdf]

Supplementary Figures

Figure S1 EBV sequence read depth and contig maps.

EBV sequence reads were aligned to the reference type 1 (NC007605) or type 2 NC009334) genome and read depths displayed in Macvector. The contigs obtained from the SPAdes assembly are represented by the lines beneath each panel. The deletions in the EBV genome are shown by the red blocks. Systematic over or under representation of reads in some parts of the genome in all samples are due to sequence repeat arrays in the virus genome and varying efficiency of the hybrid enrichment oligonucleotides.

Figure S2 Phylogenetic tree comparing the 21 new EBV genomes with published EBV genomes.

The EBV genomes from patients 1-21 (T/NK) and 241 EBV genomes (C) described previously (22) were analysed using the MEGA7 maximum likelihood method (41), with the bootstrap consensus tree inferred from 500 replicates. Geographic origins of samples are shown with colours and the 21 new samples are offset for clarity. The new India, Pakistan, Sri Lanka group is indicated.

Figure S3 Group of SNPs unique to EBV genomes from India, Pakistan, Sri Lanka.

Figure S4 PCR primers used for amplifying across EBV genome deletions.

Figure S5

(A) BVRF1 and BVLF1 recoding alignment in BAC plasmid used for cloning Jijoye EBV.

(B) Characterisation of Jijoye BAC clone by pulsed field electrophoresis.

Figure S1

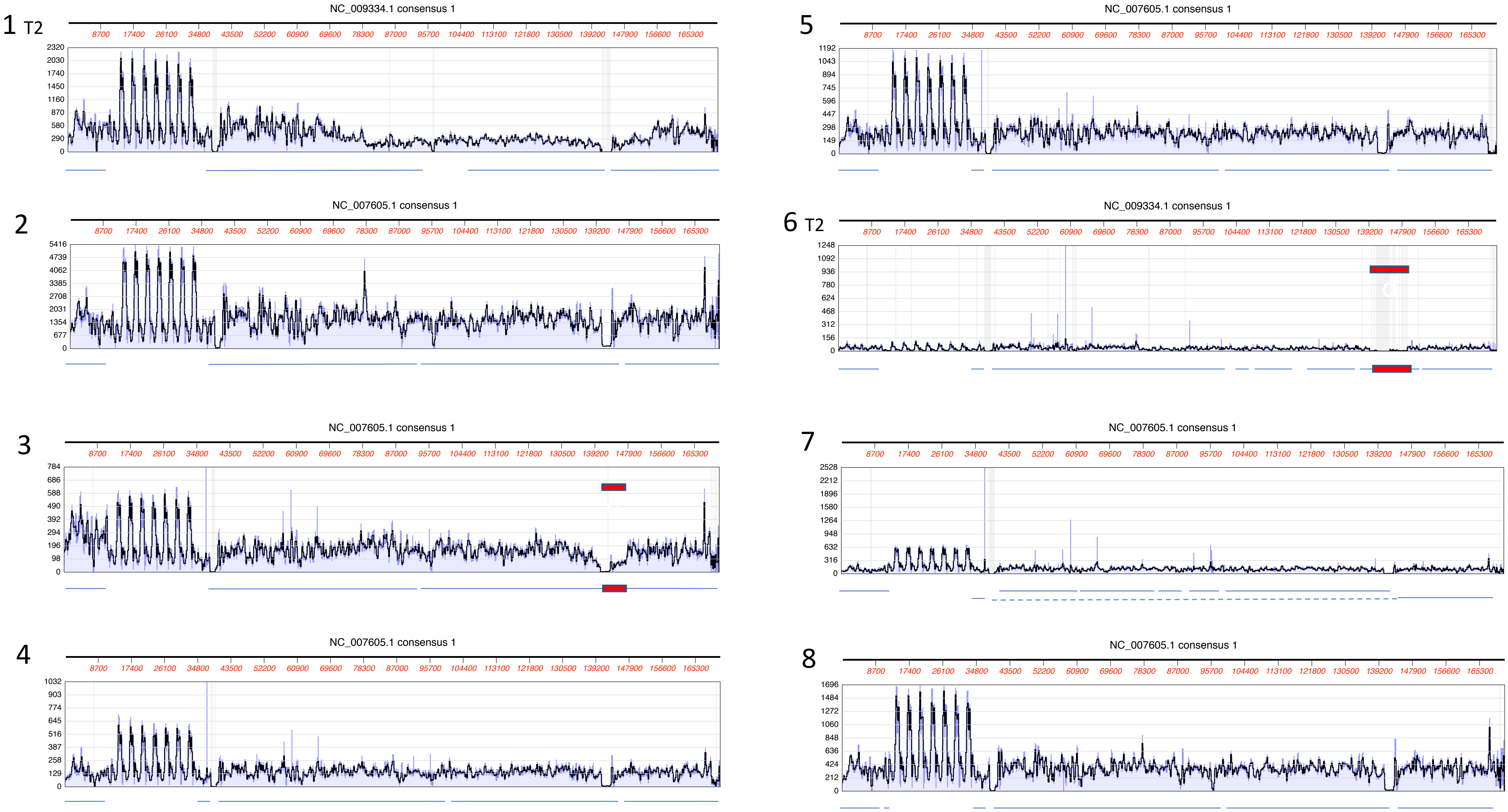
1 T2
NC_009334.1 consensus 1
8700 17400 26100 34800 43500 52200 60900 69600 78300 87000 95700 104400 113100 121800 130500 139200 147900 156600 165300
2320 2030 1740 1450 1160 870 580 290 0
2
NC_007605.1 consensus 1
8700 17400 26100 34800 43500 52200 60900 69600 78300 87000 95700 104400 113100 121800 130500 139200 147900 156600 165300
5416 4739 4062 3385 2708 2031 1354 677 0
3
NC_007605.1 consensus 1
8700 17400 26100 34800 43500 52200 60900 69600 78300 87000 95700 104400 113100 121800 130500 139200 147900 156600 165300
784 686 588 490 392 294 196 98 0
4
NC_007605.1 consensus 1
8700 17400 26100 34800 43500 52200 60900 69600 78300 87000 95700 104400 113100 121800 130500 139200 147900 156600 165300
1032 903 774 645 516 387 258 129 0
5
NC_007605.1 consensus 1
8700 17400 26100 34800 43500 52200 60900 69600 78300 87000 95700 104400 113100 121800 130500 139200 147900 156600 165300
1192 1043 894 745 596 447 298 149 0
6 T2
NC_009334.1 consensus 1
8700 17400 26100 34800 43500 52200 60900 69600 78300 87000 95700 104400 113100 121800 130500 139200 147900 156600 165300
1248 1092 936 780 624 468 312 156 0
7
NC_007605.1 consensus 1
8700 17400 26100 34800 43500 52200 60900 69600 78300 87000 95700 104400 113100 121800 130500 139200 147900 156600 165300
2528 2212 1896 1580 1264 948 632 316 0
8
NC_007605.1 consensus 1
8700 17400 26100 34800 43500 52200 60900 69600 78300 87000 95700 104400 113100 121800 130500 139200 147900 156600 165300
1696 1484 1272 1060 848 636 424 212 0

9

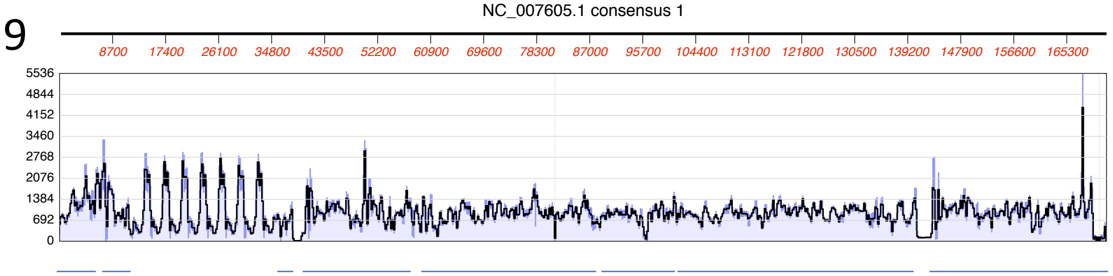

10

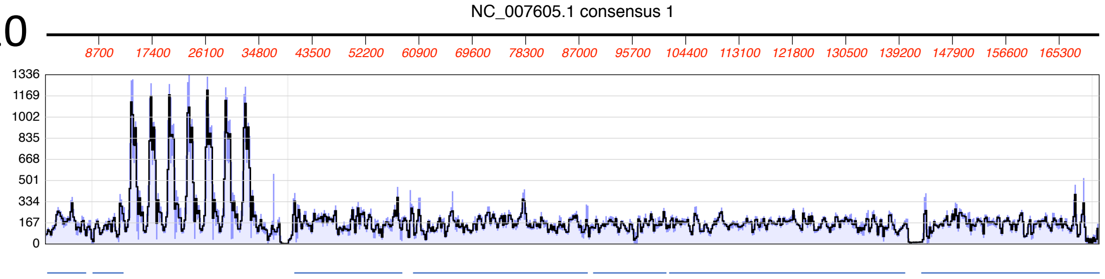

11

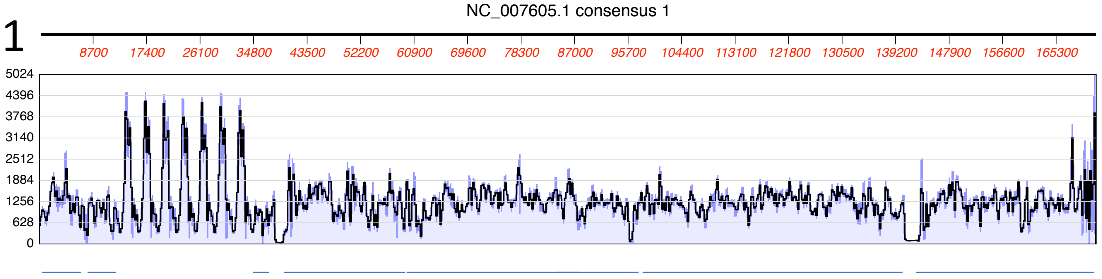

12

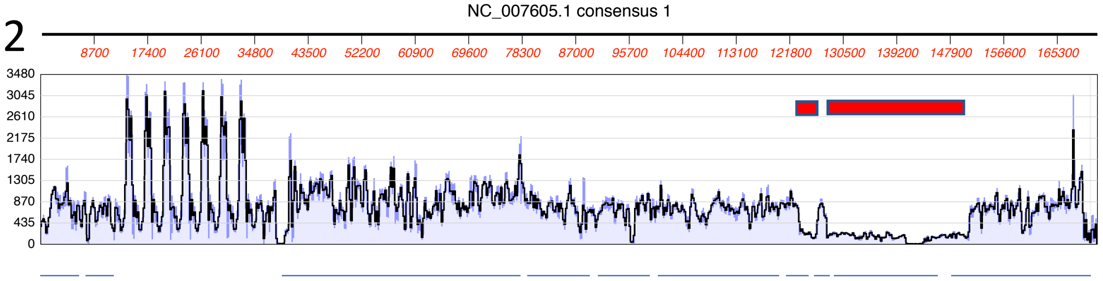

13

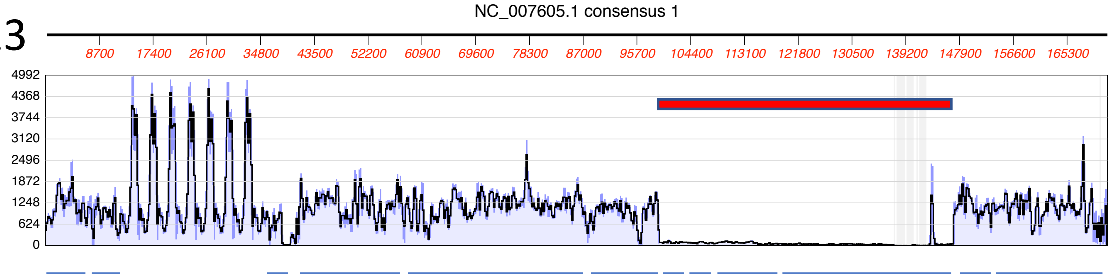

14

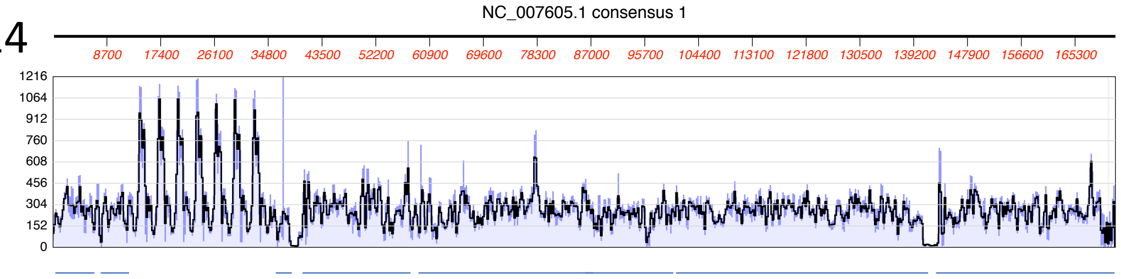

15

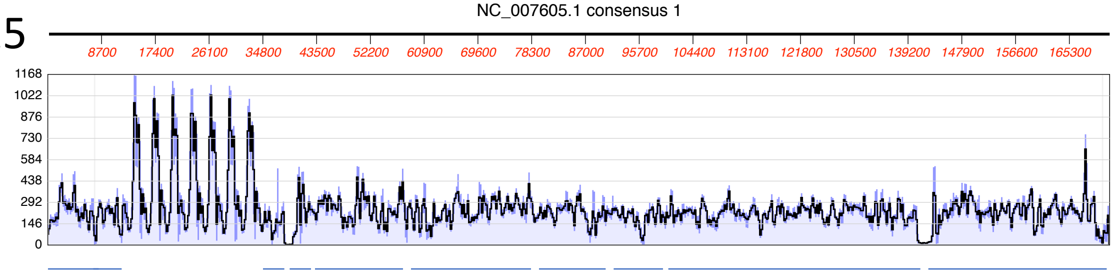

16 T2

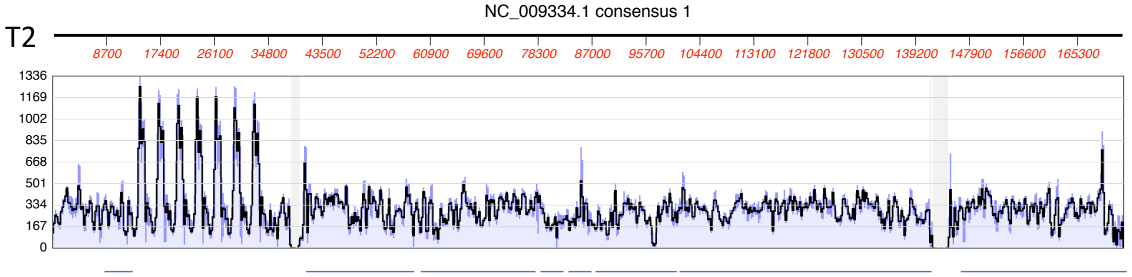

17

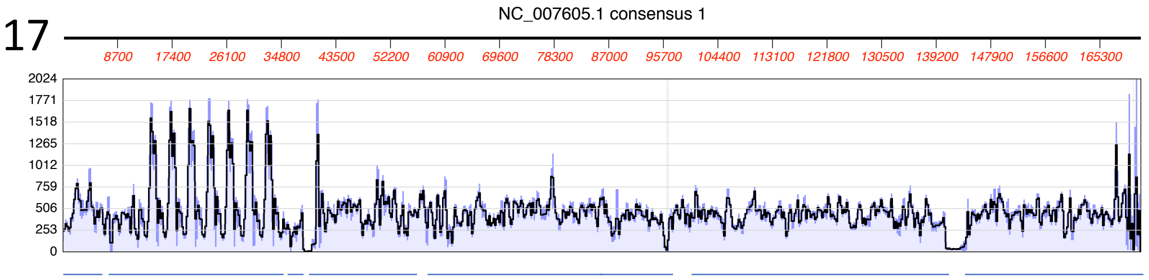
NC_007605.1 consensus 1
8700 17400 26100 34800 43500 52200 60900 69600 78300 87000 95700 104400 113100 121800 130500 139200 147900 156600 165300
2024 1771 1518 1265 1012 759 506 253 0

18

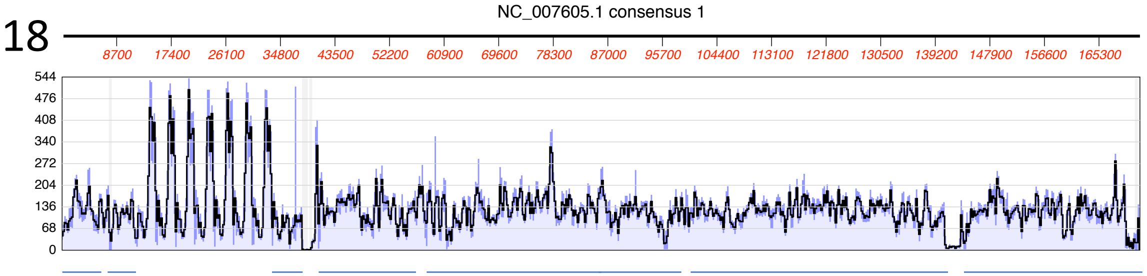
NC_007605.1 consensus 1
8700 17400 26100 34800 43500 52200 60900 69600 78300 87000 95700 104400 113100 121800 130500 139200 147900 156600 165300
544 476 408 340 272 204 136 68 0

19

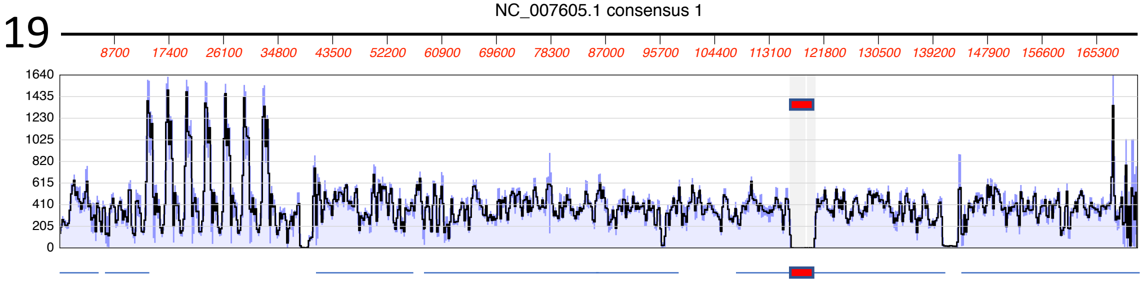
NC_007605.1 consensus 1
8700 17400 26100 34800 43500 52200 60900 69600 78300 87000 95700 104400 113100 121800 130500 139200 147900 156600 165300
1640 1435 1230 1025 820 615 410 205 0

20

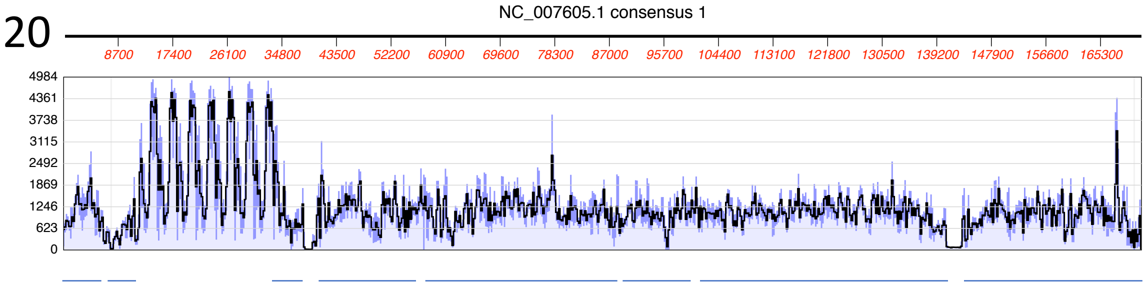
NC_007605.1 consensus 1
8700 17400 26100 34800 43500 52200 60900 69600 78300 87000 95700 104400 113100 121800 130500 139200 147900 156600 165300
4984 4361 3738 3115 2492 1869 1246 623 0

21

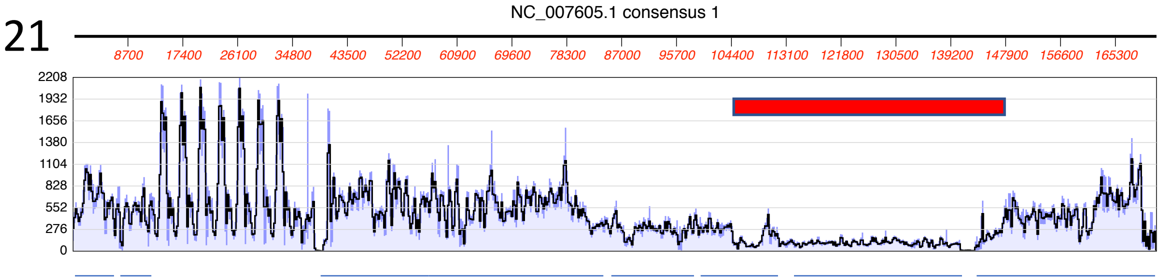
NC_007605.1 consensus 1
8700 17400 26100 34800 43500 52200 60900 69600 78300 87000 95700 104400 113100 121800 130500 139200 147900 156600 165300
2208 1932 1656 1380 1104 828 552 276 0

12

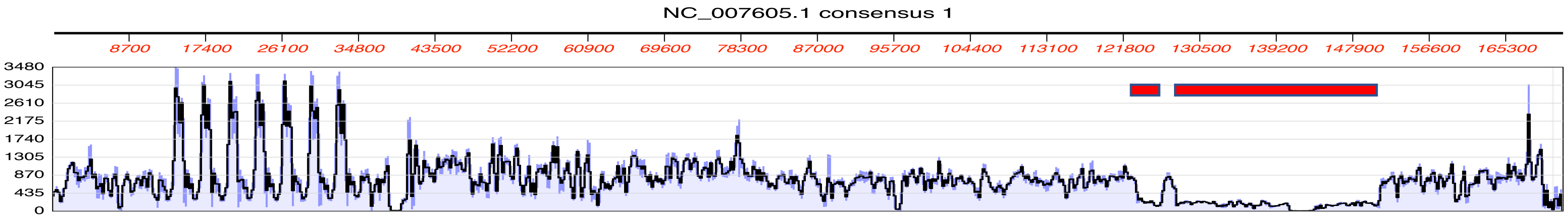

12S

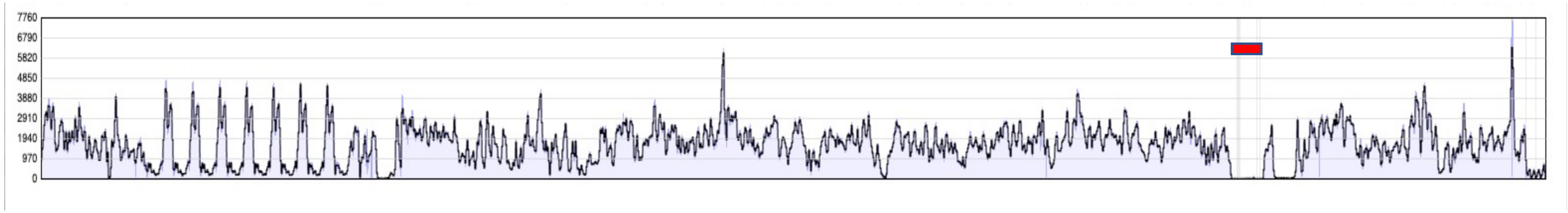

Sister of 12

12F

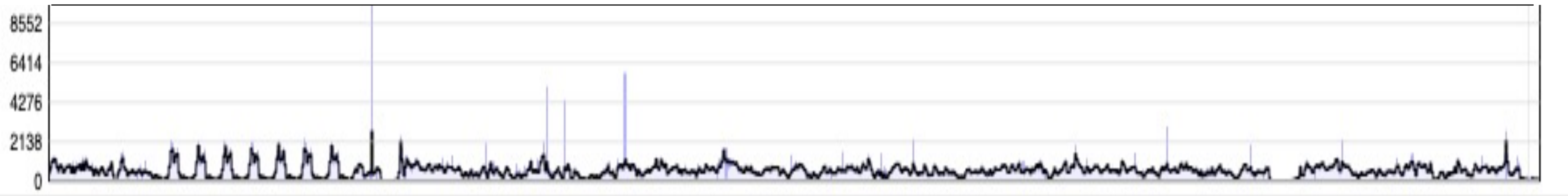

Father of 12

Figure S3

SNPs characteristic of India/Pakistan/Sri Lanka EBV strains

NC007605 position corresponding to start of sequence shown

diagnostic SNP (patient 12 sequence shown)

		Name of SNP
9655	TAGGCCTGCA**A**ACCTTAGGTA	9665C>A
71920	TGGCCGGTGT**T**GCTTCTTACC	71930A>T
74594	TATTTAAGAC**T**GGGAGGCCCC	74604C>T
77603	TTTGGGGGGG**T**GTGCCTTTGG	77613C>T
77719	GACACATGAT**A**TGTGCTGGTA	77729G>A
80148	AGCGGGTCGA**A**CATGATGCCG	80158T>A
83390	AACTAGTCCA**T**GCTGTCTATG	83400C>T
85257	ACCACCTGAG**T**TGCCGCCCGT	85267G>T
85523	AGCGTCAAGC**T**GCTGCGGGCT	85533C>T
111708	GGGTAGAGTA**T**GGTAGTTCCA	111718C>T
117597	TCGTCTCCTC**T**ATCGTCACCC	117607G>T
117882	CCTTCGATCC**A**GTCCTCTATC	117892G>A
117936	CTCTCCGCGA**T**GGGACCTACA	117946C>T

Figure S4

Primers for EBV deletions PCR

P3 -F GATAGAGACACAAGGACTGC
P3 -R GCGAAAGGCACTCCAGAAT

P6 -F ACACGAGTGAGTAGAAAGGG
P6 -R CCCTATGGCAGAAAGCAAC

P12 -F GCACAGTCTCAATAACGGC
P12 -R CTCCCTGGCTTTTCCCTAT

P13 -F ACCCAACAGTCTTCAGGTC
P13 -R GGTTCAACTCCAGGGTCTA

P19 -F GAGGTGATTGACCACAGTC
P19 -R ACCGTGAAGTTAGAGTCCA

P21D -F-D ATTTCCAGTGCCAGACCGT
P21D -R-D CCAAACTGTCTGACCCAAC

P21E F-E CCGATATAATGCCAGCCTG
P21E R-E CTCTATGACTCTGTGACGG

A12S Fwd. TCTGAAGAAGCCCTTGCCA
12S Rev. AACCTTGTATGCCTGCGTC

12 BF ATGTGGCGGTCTCAAACTG
12 BR CATTTGTGTAGGTGCGGCT

12 CF GCAGGTTACCCACCATTAG
12 CR ATCGCTCTCCCTGGCTTTT

12 F-D TGGAGGCTATTTATCCCGC
12 R-D CCTGATGCTTACACCTCGT

21 F CAAGCATTTCCAGTGCCAG
21 R CACAGGAGGCAAGTAGACA

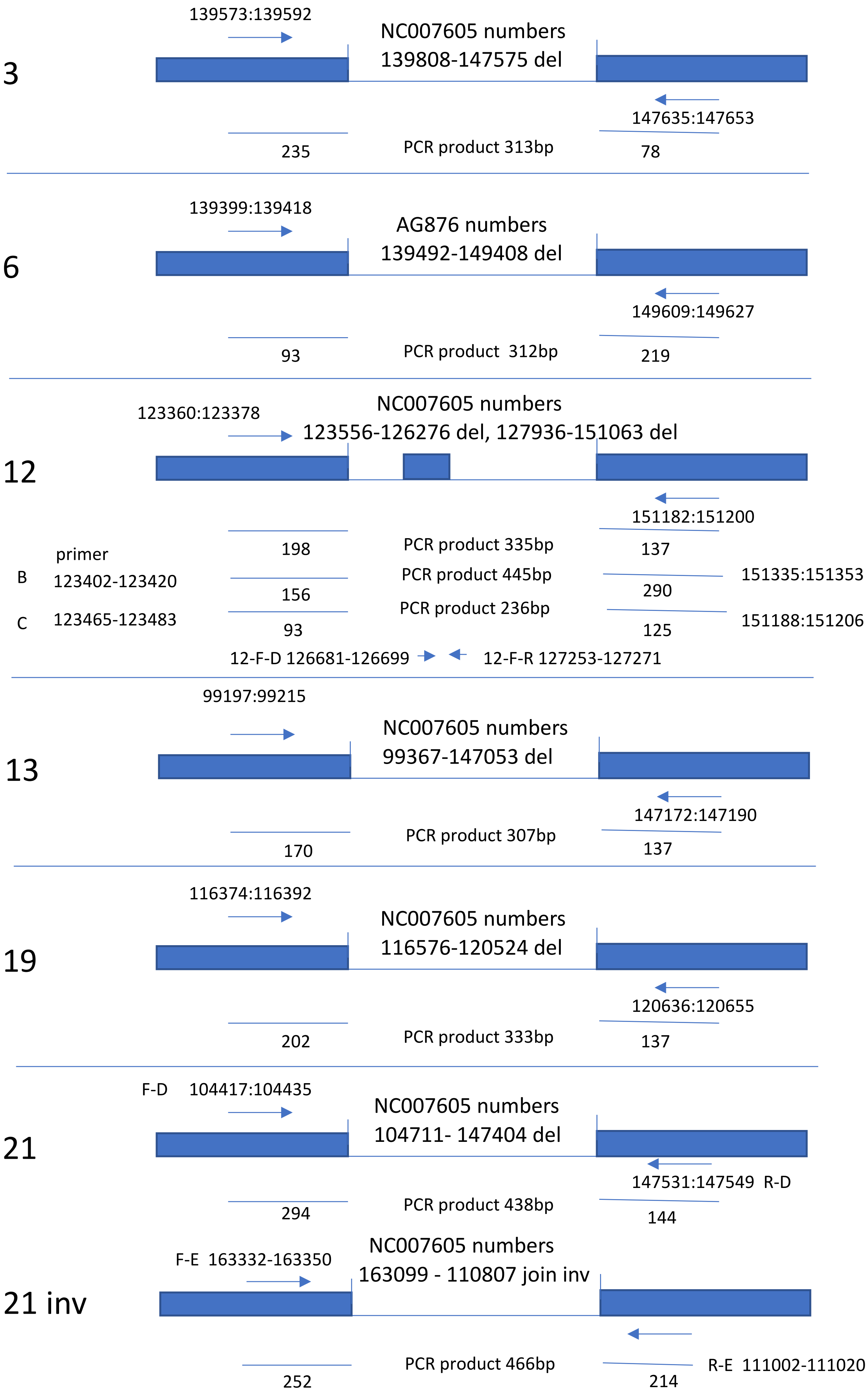

3
139573:139592
NC007605 numbers
139808-147575 del
147635:147653
235
PCR product 313bp
78
6
139399:139418
AG876 numbers
139492-149408 del
149609:149627
93
PCR product 312bp
219
12
123360:123378
NC007605 numbers
123556-126276 del, 127936-151063 del
151182:151200
198
PCR product 335bp
137
primer
B 123402-123420
156
PCR product 445bp
290
151335:151353
C 123465-123483
93
PCR product 236bp
125
151188:151206
12-F-D 126681-126699
12-F-R 127253-127271
13
99197:99215
NC007605 numbers
99367-147053 del
147172:147190
170
PCR product 307bp
137
19
116374:116392
NC007605 numbers
116576-120524 del
120636:120655
202
PCR product 333bp
137
21
F-D 104417:104435
NC007605 numbers
104711- 147404 del
147531:147549 R-D
294
PCR product 438bp
144
21 inv
F-E 163332-163350
NC007605 numbers
163099 - 110807 join inv
252
PCR product 466bp
214
R-E 111002-111020

Figure S5A

```
BVRF1 codons ->                 H  Y  A  I  N  L  T  G  Q  K  F  D  T  L  F  E  I  I  N  Q
  BVRF1 recoded                CATTATGCCATAAACCTGACCGGACAAAAGTTTGACACCCTCTTTGAGATTATCAACCAG
         Jijoye                CATTATGCCATAAACCTGACCGGACAAAAGTTTGACACCCTCTTTGAGATTATCAACCAG
  BVLF1 recoded                ------------------------------------------------------------

BVRF1 codons ->  K  L  L  F  H  D  P  A  A  M  L  A  A  R  T  Q  L  R  L  A  F  E  D  G  V
  BVRF1 recoded AAGCTTTTATTTCACGACCCGGCTGCCATGCTCGCCGCCCGGACCCAATTGCGCCTGGCATTTGAAGAAGGTGTG
         Jijoye AAGCTTTTATTTCACGACCCGGCTGCCATGCTGGCGGCGCGCACACAGCTGCGTCTAGCCTTCGAGGACGGCGTC
  BVLF1 recoded --------- BAC IN HERE->>-GTCGACTTAATTAACGCACACAGCTGCGTCTAGCCTTCGAGGACGGCGTC

BVRF1 codons ->  G  V  A  L  G  R  P  S  P  T  L  A  A  R  E  I  L  E  R  Q  F  S  A  S  D
  BVRF1 recoded GGAGTGGCACTTGGTCGTCCAAGCCCAACCCTGGCCGCACGAGAAATTCTGGAACGCCAGTTCAGTGCAAGTGAC
         Jijoye GGTGTTGCCCTGGGGCGCCCCTCGCCCATGCTTGCGGCGCGGGAGATCCTGGAGCGTCAGTTCTCAGCCTCGGAT
  BVLF1 recoded GGTGTTGCCCTGGGGCGGCCCTCGCCCACGCTTGCGGCGCGGGAGATCCTGGAGCGTCAATTCTCCGCCTCGGAT

BVRF1 codons ->  D  Y  D  R  L  Y  F  L  T  L  G  Y  L  A  S  P  V  A  P  S  *
  BVRF1 recoded GATTATGATCGCTTGTACTTTCTCACCTTGGGCTATCTTGCAAGCCCTGTTGCCCCATCCTAAGCCAGTTCCTCG
         Jijoye GACTACGACCGGCTGTACTTCCTGACGCTGGGCTACCTGGCCTCCCCGGTGGCCCCAAGCTGAGCCAGTTCCTCG
  BVLF1 recoded GACTACGACCGGTTGTACTTCCTGACGCTGGGTTAGCGTGCTTCACCAGTAGCACCCAGTTGCGCAAGCTCTTCA
BVLF1 codons <-                                  *  R  A  E  G  T  A  G  L  Q  A  L  E  E

  BVRF1 recoded CACTGGAGTGGGTCATTGGCAAAAAGGTAAATAAAGTCATCGCACGGGGGTTTTAATTAAGATATCGCGGCCGC-
         Jijoye CACTGGAGTGGGTCATTGGCAAAAAGGTAAATAAAGTCATCGCACGGGGGTTTTGCCTCCTTCTCGTCTCTTGTT
  BVLF1 recoded CATTGTAAGGGATCGTTCGCGAACAGATAGATGAAATCGTCACAAGGTGGCTTAGCCTCCTTCTCGTCACGTGTT
BVLF1 codons <- C  Q  L  P  D  N  A  F  L  Y  I  F  D  D  C  P  P  K  A  E  K  E  D  R  T

  BVRF1 recoded <<-BAC IN HERE-------------------------------------------------------------
         Jijoye TCGGGTAGGGGAGTAAGGCCGTGCCAGGCCGCCATGCTCAGGGCCACGGCGTGCCAGAGGCCCTCGTAGTCGTGC
  BVLF1 recoded TCGGGTAGGGGAGTAAGGCCGTGCCAGGCCGCCATGCTCAGGGCCACGGCGTGCCAGAGGCCCTCGTAGTCGTGC
BVLF1 codons <- E  P  L  P  T  L  G  H  W  A  A  M  S  L  A  V  A  H  W  L  G  E  Y  D  H
```

Figure S5A – Alignment showing the sequences of the recoded regions of BVRF1 and BVLF1 that flank the insertion of the BAC. Bold nucleotides differ from the B95-8 reference sequence. Red nucleotides have been modified to change the codon without changing the amino acid of the protein. Restriction sites into which the BAC is cloned are shown as blue nucleotides.

Figure S5B

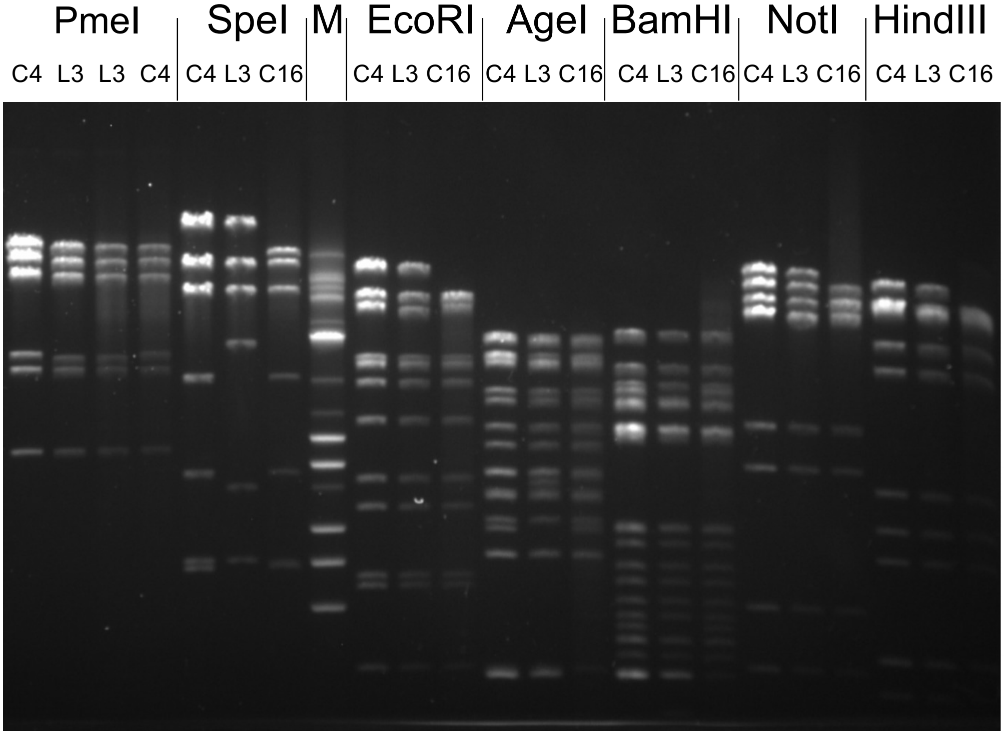

Figure S5B. Pulsed field gel analysis of restriction fragments of three BAC clones rescued from Jijoye EBV. Analysis was performed on miniprep (orange) or maxiprep (white) DNA preps. Bands match predicted sizes based on Jijoye sequence (accession number LN827800) captured by homologous recombination, except clone L3 (deletion of CMV promoter and enhance from CAG promoter) and clone C16 (deletion in W repeats).
